# High incidence of heteroplasmy in the mtDNA of a natural population of the spider crab *Maja brachydactyla*

**DOI:** 10.1371/journal.pone.0230243

**Published:** 2020-03-19

**Authors:** Elba Rodríguez-Pena, Patricia Verísimo, Luis Fernández, Ana González-Tizón, Covadonga Bárcena, Andrés Martínez-Lage

**Affiliations:** Dpto. de Biología, Facultad de Ciencias, Centro de Investigaciones Científicas Avazadas, Universidade da Coruña, A Coruña, Spain; Shanghai Ocean University, CHINA

## Abstract

Mitochondria are mostly inherited by maternal via, that is, only mitochondria from eggs are retained in the embryos. However, this general assumption of uniparentally transmitted, homoplasmic and non-recombining mitochondrial genomes is becoming more and more controversial. The presence of different sequences of mtDNA within a cell or individual, known as heteroplasmy, is increasingly reported in several taxon of animals, such as molluscs, arthropods and vertebrates. In this work, a considerable frequency of heteroplasmy were detected in the COI and 16S genes of the spider crab *Maja brachydactyla*, possibly associated to hybridisation with the congeneric species *Maja squinado*. This finding is a fact to keep in mind before addressing molecular analyses based on mitochondrial markers, since the assumption of maternal inheritance could lead to erroneous results. As *M*. *brachydactyla* is a commercial species, heteroplasmy is an important aspect to take into account for the fisheries management of this resource, since effective population size could be overestimated.

## Introduction

Mitochondria are mostly inherited by maternal via, that is, only mitochondria from eggs are retained in the embryos. Uniparental transmission of cytoplasmic genomes has had multiple and independent origins and it must involve a strong evolutionary advantage over biparental transmission [1, 2]. However, this general assumption of uniparentally transmitted, homoplasmic and non-recombining mitochondrial genomes is becoming more and more controversial [3–5]. Until now, the presence of different sequences of mtDNA within a cell or individual was a rare phenomenon in animals, but new detection methods based on NGS (Next-Generation Sequencing) or qPCR (quantitative Polymerase Chain Reaction) are allowing their detection [6–9]. Nowadays, heteroplasmy has been reported in many organisms such as insects [10, 11], crustaceans [12, 13], molluscs [14], fishes [15, 16], frogs [17] birds [18, 19], mice [20] and humans [21, 22], being the paternal leakage the primary cause of it. The existence of heteroplasmy in the studied organisms can provide erroneous results in researches that use mitochondrial markers. This factor must be taken into consideration before interpreting the obtained results. For example, the assumption of maternal mitochondrial inheritance in species with multiple mtDNAs per individual can result in an estimation of the effective population size larger than it really is as well as create false negatives in lineage determination.

There may be five main reasons for the presence of heteroplasmy [23]:

- occurrence of a *de novo* mutation in somatic line or germ line cells. In the first case, heteroplasmy is not transmitted to the offspring, while in the second case, it is. Studies in the crustacean *Daphnia pulex* estimate the frequency of *de novo* mutations at 1.63 x 10^−7^ / site / generation [24].
- recombination events. However, despite the high number of mitochondrial molecules that exist in an individual, the possibility of being able to detect this type of heteroplasmy is limited since its frequency is usually very low. In humans, recombination intermediates were detected in the mtDNA, varying their abundance between tissues (high in heart, intermediate in skeletal muscle or placenta, but low or absent in cultured cells) [25].
- paternal leakage as it has been reported in fruit fly [11] and hen [26]. There are several mechanisms for preventing the transmission of paternal mtDNA to offspring in animals: (a) degradation of paternal mtDNA before or after fertilisation, (b) blocking paternal mitochondria from entering the oocyte, (c) elimination of paternal mitochondria by autophagy and/or ubiquitin-proteasome systems and (d) uneven distribution of paternal mitochondria with remaining paternal mtDNA during embryogenesis [27]. In the nematode *Caenorhabditis elegans*, paternal mitochondria and their mtDNA degenerate almost immediately after fertilisation and are selectively degraded by ‘allophagy’ (allogeneic [non-self] organelle autophagy) [28]. In the fruit fly *Drosophila melanogaster*, paternal mtDNA is largely eliminated by an endonuclease G-mediated mechanism. Paternal mitochondria are subsequently removed by endocytic and autophagic pathways after fertilisation [29]. The reason of the existence of mechanisms to prevent paternal mtDNA transmission is not well understood yet. The most plausible explanation is that the retaining of two different, but individually fully functional, mtDNAs within a cell can cause mitochondrial dysfunction due to a potentially lethal genome conflict [30, 31]. Nevertheless, it has also been argued as an adaptation to anisogamy, which prevents sperm mtDNA, damaged from intense respiration activity, entering the egg [32]. Nevertheless, in some cases, a breakdown of mechanisms to recognise and remove paternal mtDNA may occur resulting in paternal leakage.
- biparental inheritance. The concepts of parental leakage and biparental inheritance may not be clearly delimited, depending on the definition of the second one. While some authors consider incidental paternal leakage constitutes a form of biparental mtDNA transmission, Breton and Stewart [23] define "true" biparental transmission as the systematic transfer of mitochondrial genomes from both parents (or two different mating types) to zygotes as part of normal reproductive processes within a species, followed by the persistence of both parental mitochondrial types throughout development. So far, following this narrow definition, it has not been reported any organism that present this mode of biparental mtDNA inheritance.
- doubly uniparental inheritance (DUI), typically observed in some bivalvian molluscs [2, 33–36]. Many bivalves have a sperm-transmitted mitochondrial genome (M), along with the standard egg-transmitted one (F). During embryonic development of the mussel *Mytilus*, sperm mitochondria disperse randomly among blastomeres in females, but form an aggregate in the same blastomere in males. Consequently, in adults, somatic tissues of both sexes are dominated by the F mitochondrial genome, sperm contains only the M genome, and eggs contain the F genome (and perhaps traces of M).

In addition, events of duplications of some mitochondrial genes [33] or the formation of nuclear-encoded, mitochondrial pseudogenes (NUMTs) [33, 37] may also be interpreted erroneously as heteroplasmy. NUMTs are described as a transposition of mitochondrial DNA into the nuclear genome that can retain close homology to the original mitochondrial genes [38].

The spiny spider crab *Maja brachydactyla* (Balss 1922) [39] is a decapod crustacean of the Majidae family [40,41] very common on the European Atlantic coast. This species was initially classified within the species *Maja squinado* (Herbst 1788) [42], but in recent years morphological [43, 44] and molecular [45] differences between the Atlantic and Mediterranean populations have confirmed that it is a different species. After this separation, its distribution is considered to be limited to the Eastern Atlantic, from the South of the North Sea to South Africa, including the islands of Madeira, Azores, Canary Islands and Cape Verde [44, 46–49].

This species presents a complex life cycle, with planktonic larval and benthic post-larvae phases, which determines that its distribution and population dynamics are closely related to larval dispersion processes, mediated by physical factors, as well as habitat selection, linked to movements and migrations in post-larval stages [50, 51]. The life cycle of *M*. *brachydactyla* consists of three main phases: larval phase, juvenile or growth phase, and adult or reproductive phase. The planktonic larval phase lasts from two to three weeks and consists of two zoeal stages (zoea I and II) and one megalopa stage [52, 53]. The juvenile phase lasts from two to three years [54], inhabiting shallow bottoms (<15m) where they perform limited, non-directional, small-scale movements of less than 10 m per day [47, 55, 56]. Their growth occurs through successive moults and, after terminal moult, which takes place in spring and summer, individuals reach sexual maturity and begin the adult phase.

*M*. *brachydactyla* is a species of high commercial interest in several countries, especially in Spain. Galicia (a region of NW Spain), with around 300 vessels devoted to spider crab fishing, represents the main exploitation region of the country, both in terms of production (73%) and incomes (more than 80%). In 2018, the captures of *M*. *brachydactyla* reached the 525934 kg in Galician coast, which represented around 4640000 euros in sales [57].

During the course of a study with mitochondrial molecular markers in *M*. *brachydactyla*, double peaks were detected in some of the electropherograms obtained. The studies by Abelló *et al*. [58] already suggested the possible existence of heteroplasmy in this species. The present work confirms the existence of heteroplasmy in the spiny spider crab and delves into this aspect to determine the magnitude and possible origin of this phenomenon.

## Material and methods

Specimens of *M*. *brachydactyla* were captured in two locations of the Galician coast, NW Spain: Golfo Ártabro (43°25'N 8°21'W) and Ría de Arousa (42°33'N 8°54'W). Most of the spider crabs were captured by the scuba divers of the Aquarium Finisterrae of A Coruña (Spain) with a permit emitted by the Consellería do Medio Rural e do Mar of the Xunta de Galicia (regional government, resolution of 18/02/2014). The rest of the specimens were directly bought at the fish market. Since *M*. *brachydactyla* is a commercial species, no protected species were sampled during this study, and sample collection has not been conducted in a protected or privately owned area. All the specimens were anesthetized at -20°C for 10 min before being slaughtered. Regarding the Golfo Ártabro, pereiopod muscle from 27 males and pleopod setae from 23 females were collected. Fertilised eggs from 11 of these females were also sampled (2–17 eggs depending on the female). Furthermore, different tissues and organs from four of these females from Golfo Ártabro (pleopod setae, integument, eye, pereiopod muscle, gonad, heart, nervous tissue, hepatopancreas, stomach, gill, intestine, pereiopod setae and mouth-parts) were collected. In addition to this, gonadal tissue were sampled from 33 females from Ría de Arousa. All samples were preserved in absolute ethanol until the time of DNA extraction. The sampling of individuals of both sexes and broods of known females was performed in order to determine the origin and the inheritance mechanism of the heteroplasmy in *M*. *brachydactyla*. The analysis of different tissues of the same individual provides information about the distribution of the mtDNA sequences in early development stages and the extension of the heteroplasmy in the different tissues in adults.

DNA extraction was conducted using the NZY Tissue gDNA Isolation kit (nzytech, Portugal). All the laboratory material used in this process (scissors, tweezers, pestles) was washed in absolute ethanol between consecutive samples. Two different mitochondrial DNA gene fragments were amplified by PCR: the mitochondrial 16S rDNA and the cytochrome c oxidase subunit I (COI). While COI was analysed in all the samples, 16S was only sequenced for the 11 ovigerous females from Golfo Ártabro and their eggs. The PCR conditions for the amplification of 16S rDNA were described in Rodríguez-Pena *et al*. [59]. COI fragment was amplified using the primers COIMaja_F 5´-gaatggccggaacatcttta-3´, and COIMaja_R 5´-ccaccagctggatcaaagaa-3´ and the NZYTaq 2xGreen Master Mix separate MgCl_2_ kit (NZYTech) according to the manufacturer´s protocol in a final concentration of 1.5 mM of MgCl_2_ and 0.5μM of each primer. The PCR consisted in a denaturalization step of 5 min at 94°C, 35 cycles of 30 s at 94°C, 30 s at 48.5°C and 60 s at 72°C, and a final extension of 5 min at 72°C. All PCRs yielded single-band patterns, so that the resultant amplicons were directly sequenced in both directions.

Some of the PCR products of 16S rDNA were cloned using StrataClone PCR Cloning Kit according to the manufacturer´s protocol in order to determine the number of nucleotide combinations present in heteroplasmic samples. Cells were spread on an LB-ampicillin plate and let it grow overnight at 37°C. Transformant colonies were selected, and insert size was checked by PCR. Plasmids were purified using the NZYMiniprep kit (nzytech, Portugal), and they were sequenced using the M13 forward and reverse primers. The PCR consisted in a denaturalization step of 5 min at 94°C, 35 cycles of 60 s at 94°C, 60 s at 55°C, 60 s at 72°C, and a final extension of 5 min at 72°C.

The sequencing profiles were examined using BioEdit 7.0.9.0 [60]. In order to detect the presence of double peaks in the PCR products, reads in both directions were checked. We repeated the DNA extractions, amplifications and sequencing to verify these double peaks were not due to errors in the PCRs. The frequency of each nucleotide was estimated as the average of the peaks of the two reads. From the unambiguous sequences, a consensus sequence was established. Next, all the amplicons were aligned using the tool Muscle [61] of MEGA 6.06 [62]. The variable sites, the haplotypes and their frequencies were calculated using the DnaSP 6.10.04 [63] and the mitochondrial genetic code of *Drosophila*.

## Results

**Fig 1:**
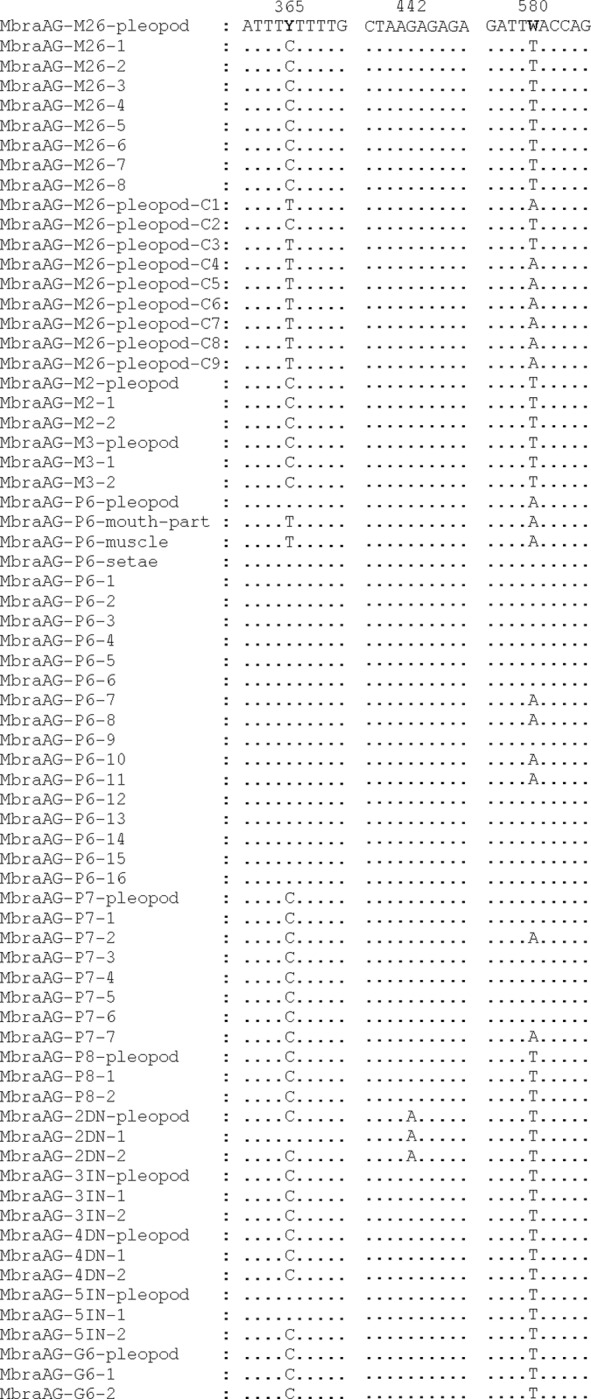
Alignment of the obtained sequences of 16S gene around positions 365, 442 and 580. The sequences were named with the following codes: MbraAG-specimen code-tissue, in the case of adults; MbraAG-female code-number, in the case of the eggs. Dots indicate identity with the consensus sequence (on top).

The sequence alignments of 16S mtDNA allowed us to detect the existence of three variable sites: 365, 442 and 580. Two of them are parsimony informative (365 and 580), while 442 is a singleton. Table 1 (GenBank accession numbers: MN006155—MN006157) shows the three different haplotypes detected. We identified double peaks in the electropherograms of 16S mtDNA of the following adult females: MbraAG-M26 (at positions 365 and 580), MbraAG-P6 and MbraAG-5IN (at position 365), and MbraAG-P7 (at position 580) (Table 2 and Fig 1). After repeating DNA extractions, PCRs and sequencing, we detected again these ambiguous sites at the same positions of the same samples.

**Table 1:** Detected haplotypes for the 16S gene and adult individuals in which they were observed.

Haplotype	N	Individual			
Hap-16S-1	8	MbraAG-M2-pleopod	MbraAG-M3-pleopod	MbraAG-P7-pleopod	MbraAG-P8-pleopod
		MbraAG-3IN-pleopod	MbraAG-4DN-pleopod	MbraAG-5IN-pleopod	MbraAG-G6-pleopod
Hap-16S-2	1	MbraAG-2DN-pleopod			
Hap-16S-3	2	MbraAG-M26-pleopod	MbraAG-P6-pleopod		
Total	11				

N: number of haplotypes. Sequences with double peaks were not included in this analysis.

**Table 2:** Nucleotidic proportions (%) at positions 365 and 580 of the amplified mitochondrial 16S rDNA fragment for all the samples analysed.

Position		365		580	
Sample	Origin	T	C	T	A
MbraAG-M2-pleopod	female	0	100	100	0
MbraAG-M2-1	egg	0	100	100	0
MbraAG-M2-2	egg	0	100	100	0
MbraAG-M3-pleopod	female	0	100	100	0
MbraAG-M3-1	egg	0	100	100	0
MbraAG-M3-2	egg	0	100	100	0
**MbraAG-M26-pleopod**	**female**	**78.4**	**21.6**	**19.4**	**80.6**
MbraAG-M26-1	egg	0	100	100	0
MbraAG-M26-2	egg	0	100	100	0
MbraAG-M26-3	egg	0	100	100	0
MbraAG-M26-4	egg	0	100	100	0
MbraAG-M26-5	egg	0	100	100	0
MbraAG-M26-6	egg	0	100	100	0
MbraAG-M26-7	egg	0	100	100	0
MbraAG-M26-8	egg	0	100	100	0
MbraAG-M26-pleopod-C1	clone female	100	0	0	100
MbraAG-M26-pleopod-C2	clone female	0	100	100	0
MbraAG-M26-pleopod-C3	clone female	100	0	100	0
MbraAG-M26-pleopod-C4	clone female	100	0	0	100
MbraAG-M26-pleopod-C5	clone female	100	0	0	100
MbraAG-M26-pleopod-C6	clone female	100	0	0	100
MbraAG-M26-pleopod-C7	clone female	100	0	0	100
MbraAG-M26-pleopod-C8	clone female	100	0	0	100
MbraAG-M26-pleopod-C9	clone female	100	0	0	100
**MbraAG-P6-pleopod**	**female**	**93.5**	**6.5**	**0**	**100**
MbraAG-P6-mouth-part	female	100	0	0	100
MbraAG-P6-muscle	female	100	0	0	100
**MbraAG-P6-setae**	**female**	**12.2**	**87.8**	**80.6**	**19.4**
MbraAG-P6-1	egg	87.3	12.7	15.5	84.5
MbraAG-P6-2	egg	65.9	34.1	26.1	73.9
MbraAG-P6-3	egg	85.0	15.0	11.1	88.9
MbraAG-P6-4	egg	68.0	32.0	26.3	73.7
MbraAG-P6-5	egg	68.7	31.3	29.1	70.9
MbraAG-P6-6	egg	74.1	25.9	19.9	80.1
MbraAG-P6-7	egg	91.4	8.6	0	100
MbraAG-P6-8	egg	90.2	9.8	0	100
MbraAG-P6-9	egg	57.6	42.4	83.6	16.4
MbraAG-P6-10	egg	92.9	7.1	0	100
MbraAG-P6-11	egg	90.6	9.4	0	100
MbraAG-P6-12	egg	87.2	12.8	9.8	90.2
MbraAG-P6-13	egg	75.8	24.2	21.8	78.2
MbraAG-P6-14	egg	72.9	27.1	23.9	76.1
MbraAG-P6-15	egg	83.7	16.3	12.5	87.5
MbraAG-P6-16	egg	75.1	24.9	22.2	77.8
**MbraAG-P7-pleopod**	**female**	**0**	**100**	**43.5**	**56.5**
MbraAG-P7-1	egg	0	100	28.0	72.0
MbraAG-P7-2	egg	0	100	0	100
MbraAG-P7-3	egg	0	100	57.9	42.1
MbraAG-P7-4	egg	0	100	64.5	35.5
MbraAG-P7-5	egg	0	100	35.7	64.3
MbraAG-P7-6	egg	0	100	19.8	80.2
MbraAG-P7-7	egg	0	100	0	100
MbraAG-P8-pleopod	female	0	100	100	0
MbraAG-P8-1	egg	0	100	100	0
					
MbraAG-2DN-pleopod	female	0	100	100	0
MbraAG-2DN-1	egg	16.7	83.3	100	0
MbraAG-2DN-2	egg	0	100	100	0
MbraAG-3IN-pleopod	female	0	100	100	0
MbraAG-3IN-1	egg	0	100	100	0
MbraAG-3IN-2	egg	0	100	100	0
MbraAG-4DN-pleopod	female	0	100	100	0
MbraAG-4DN-1	egg	0	100	100	0
MbraAG-4DN-2	egg	0	100	100	0
**MbraAG-5IN-pleopod**	**female**	**19.2**	**80.8**	**100**	**0**
MbraAG-5IN-1	egg	10.3	89.7	100	0
MbraAG-5IN-2	egg	0	100	100	0
MbraAG-G6-pleopod	female	0	100	100	0
MbraAG-G6-1	egg	0	100	100	0
MbraAG-G6-2	egg	0	100	100	0

Proportions were calculated by dividing the height of each peak in the electropherogram by the summation of the height of both peak. The eggs and the clones correspond to the female that precedes them in the list. All samples tagged as “pleopod” correspond to pleopod setae from adult females. The samples MbraAG-P6-pleopod, MbraAG-P6-mouth-part, MbraAG-P6-muscle and MbraAG-P6-setae, correspond respectively to pleopod setae, one endite, pereiopod muscle and pereiopod setae of the same female. Adult heteroplasmic individuals are indicated in bold.

Regarding the fertilised eggs of these 11 ovigerous females, we detected heteroplasmy in all the eggs of MbraAG-P6, in five out of seven of MbraAG-P7 and in one out of two of MbraAG-5IN (Table 2) at least at one of the two positions (365 and/or 580). In addition to this, a double peak was also detected at position 365 of the MbraAG-2DN-1 egg, in whose mother no type of polymorphism were previously detected.

Due to the existence of double peaks at positions 365 and 580, two to four combinations of different mitochondrial molecules may be present. To clarify this aspect, the PCR products of the pleopod setae of the female MbraAG-M26, in which the double peaks were well-defined, were cloned and sequenced. In this case, it was detected that at least three different mitochondrial combinations are present in the pleopod setae of this female (Table 2).

**Fig 2:**
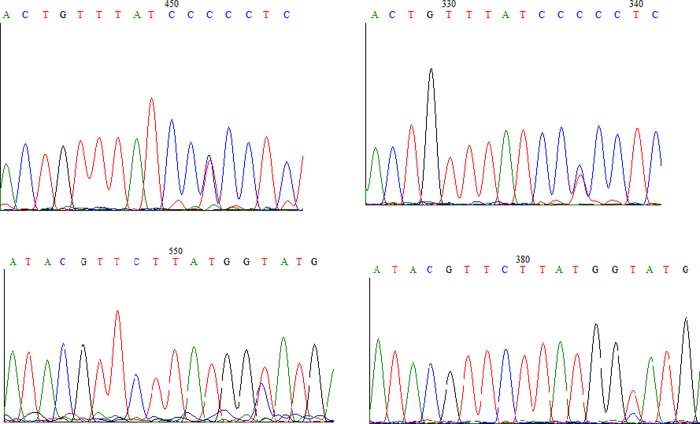
Example of double peaks in the forward (left) and reverse (right) reads of the COI region at positions 240 (top) and 375 (bottom).

The next step involved to perform a more extensive analysis of the degree of heteroplasmy in Galician populations. In order to achieve that, we used the COI sequences obtained from 50 adult individuals from Golfo Ártabro and 33 from Ría de Arousa to establish a consensus sequence, since the level of genetic variation in this mtDNA region is higher than in 16S rDNA. The electropherograms of the 83 studied individuals were checked in search of double peaks (Fig 2). After removing the sequences with ambiguous sites (double peaks), an alignment was performed with the remaining 61 sequences, which allowed to verify the existence of 26 haplotypes (Table 3, GenBank accession numbers: MN027519—MN027544). Thus, the existence of 25 variable sites was tested, of which 12 were singleton variable sites, while 13 were parsimony informative sites. No stop codons were detected in any case, being five the maximum number of synonym differences between two sequences and being four the maximum number of non-synonym differences.

**Table 3:** Detected haplotypes for the COI gene and adult individuals in which they were observed.

Haplotype	N	Individual		
Hap-COI-1	2	MbraAG-5TM-muscle	MbraAG-16TM-muscle	
Hap-COI-2	1	MbraAG-13TM-muscle		
Hap-COI-3	7	MbraAG-14H-muscle	MbraAG-15TM-muscle	MbraAG-17H-muscle
		MbraAG-23TM-muscle	MbraRA-G8-gonad	MbraRA-G46-gonad
		MBraRA-G53-gonad		
Hap-COI-4	1	MbraAG-10TM-muscle		
Hap-COI-5	1	MbraAG-18H-muscle		
Hap-COI-6	1	MBraRA-G47-gonad		
Hap-COI-7	1	MbraAG-22TM-muscle		
Hap-COI-8	1	MbraRA-G20-gonad		
Hap-COI-9	2	MbraAG-3TM-muscle	MbraRA-G21-gonad	
Hap-COI-10	1	MbraAG-2DN-pleopod		
Hap-COI-11	3	MbraAG-3H-muscle	MbraAG-20TM-muscle	MbraRA-G30-gonad
Hap-COI-12	2	MbraAG-11H-muscle	MbraAG-13H-muscle	
Hap-COI-13	15	MbraAG-2H-muscle	MbraAG-4H-muscle	MbraAG-6H-muscle
		MbraAG-7H-muscle	MbraAG-9H-muscle	MbraAG-12TM-muscle
		MBraAG-14TM-muscle	MbraAG-19TM-muscle	MbraAG-19H-muscle
		MbraAG-27TM-muscle	MbraRA-G26-gonad	MbraRA-G33-gonad
		MbraRA-G34-gonad	MbraRA-G39-gonad	MbraRA-G50-gonad
Hap-COI-14	1	MbraAG-26TM-muscle		
Hap-COI-15	1	MBraAG-28TM-muscle		
Hap-COI-16	1	MbraAG-4TM-muscle		
Hap-COI-17	3	MbraAG-16H-muscle	MbraRA-G18-gonad	MBraRA-G29-gonad
Hap-COI-18	1	MbraAG-25TM-muscle		
Hap-COI-19	7	MbraAG-6TM-muscle	MbraAG-15H-muscle	MbraRA-G5-gonad
		MbraRA-G9-gonad	MbraRA-G16-gonad	MbraRA-G23-gonad
		MBraRA-G36-gonad		
Hap-COI-20	2	MbraRA-G10-gonad	MbraRA-G35-gonad	
Hap-COI-21	1	MBraAG-18TM-muscle		
Hap-COI-22	2	MbraAG-20H-muscle	MbraRA-G43-gonad	
Hap-COI-23	1	MBraRA-G40-gonad		
Hap-COI-24	1	MbraAG-7TM-muscle		
Hap-COI-25	1	MbraAG-17TM-muscle		
Hap-COI-26	1	MbraAG-24TM-muscle		
Total	61			

N: number of haplotypes. Sequences with double peaks were not included in this analysis. Samples tagged as “pleopod” and “muscle” correspond to pleopod setae and muscle from pereiopod, respectively.

**Fig 3:**
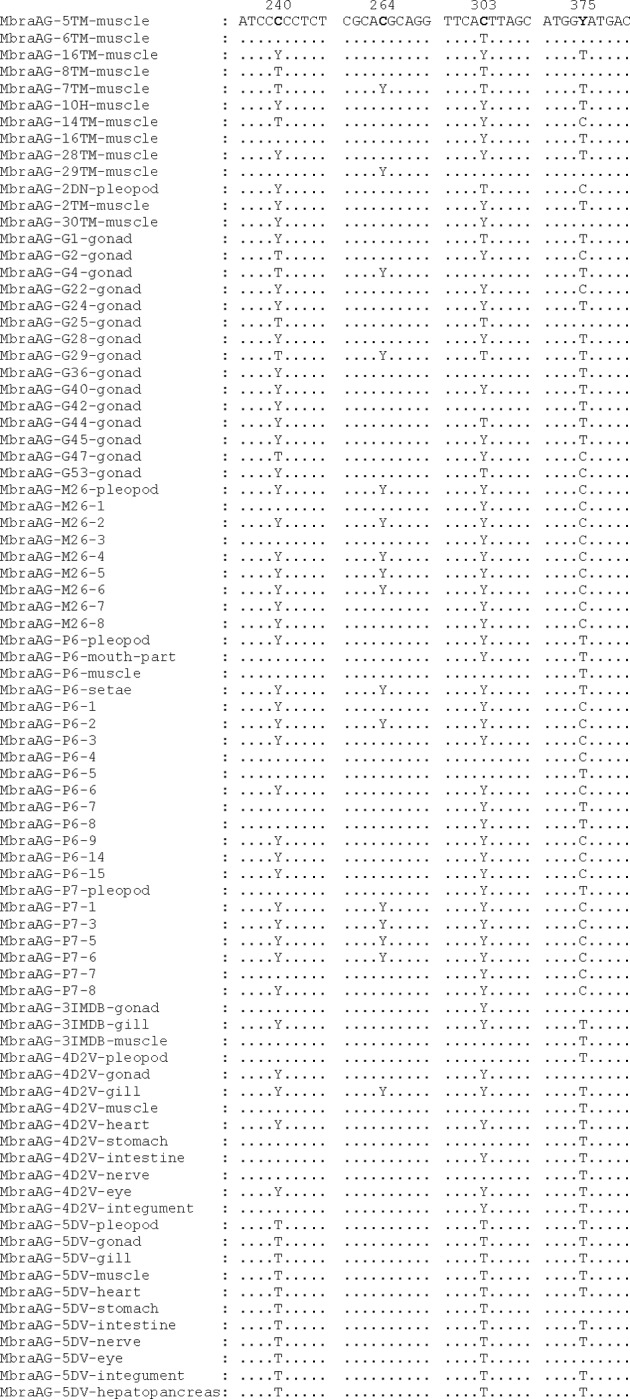
Alignment of the obtained sequences of COI gene around positions 240, 264, 303 and 375. The sequences were named with the following codes: MbraAG-specimen code-tissue, in the case of adults; MbraAG-female code-number, in the case of the eggs. Dots indicate identity with the consensus sequence (on top).

The electropherograms of the 13 parsimony informative sites were analysed at four positions (240, 264, 303 and 375) because of their variability in the sequences and the production of synonymous changes. Thus, a total of 22 individuals (four males and seven females from the Golfo Ártabro, 11 females from the Ría de Arousa) showed double peaks at one of these four positions (Table 4 and Fig 3). After repeating the DNA extraction and COI amplification, double peaks were detected at the same positions. These double peaks were only taken into consideration when the electropherograms were well-defined and there was a clear difference from the baseline. It should be highlighted that the two reads (forward and reverse) were not always identical in height due to the PCR yield may vary depending on the hybridisation of the primers and the polymerase activity. For the rest of the nine informative positions, no clear double peaks were detected and therefore they were not taken into consideration in the analyses.

**Table 4:** Nucleotidic proportions (%) at positions 240, 264, 303 and 375 of the amplified COI fragment for 16 adult individuals with ambiguous sites were detected.

Position		240		264		303		375	
Sample	Sex	T	C	T	C	T	C	T	C
MbraAG-2TM-muscle	male	15.3	84.7	0	100	20.2	79.8	100	0
MbraAG-8TM-muscle	male	100	0	0	100	100	0	80.9	19.1
MbraAG-10H-muscle	female	26.4	73.6	0	100	31.0	69.0	100	0
MbraAG-29TM-muscle	male	0	100	74	26.3	0	100	77.3	22.7
MbraAG-30TM-muscle	male	27.1	72.9	0	100	31.3	68.7	75.8	24.2
MbraRA-G1-gonad	female	12.6	87.4	0	100	100	0	100	0
MbraRA-G2-gonad	female	100	0	0	100	78.8	21.2	0	100
MbraRA-G4-gonad	female	100	0	13	86.6	0	100	100	0
MbraRA-G22-gonad	female	17.5	82.5	0	100	19.1	80.9	0	100
MbraRA-G24-gonad	female	11.0	89.0	0	100	13.8	86.2	100	0
MbraRA-G25-gonad	female	100	0	0	100	100	0	74.7	25.3
MbraRA-G28-gonad	female	16.8	83.2	0	100	18.4	81.6	100	0
MbraRA-G40-gonad	female	40.5	59.5	0	100	42.0	58.0	100	0
MbraRA-G42-gonad	female	10.7	89.3	0	100	0	100	100	0
MbraRA-G44-gonad	female	84.2	15.8	0	100	100	0	100	0
MbraRA-G45-gonad	female	47.3	52.7	0	100	46.6	53.4	100	0

Proportions were calculated by dividing the height of each peak in the electropherogram by the summation of the height of both peaks.

The sequences of COI obtained from the broods of the females MbraAG-M26, MbraAG-P6 and MbraAG-P7 showed that a great majority of the eggs presented heteroplasmy for this gene (Table 5). All the eggs of the females MbraAG-M26 (eight eggs) and MbraAG-P7 (six eggs) presented double peaks at the some positions. In the case of the female MbraAG-P6, 9 of the 11 eggs showed ambiguous sites.

**Table 5:** Nucleotidic proportions (%) of the amplified COI fragment in broods and different tissues of several adult females.

Position		240		264		303		375	
Sample	Origin	T	C	T	C	T	C	T	C
MbraAG-M26-pleopod	female	23.3	76.7	26.4	73.6	27.6	72.4	0	100
MbraAG-M26-1	egg	0	100	0	100	25.4	74.6	0	100
MbraAG-M26-2	egg	20.8	79.2	12.0	88.0	28.6	71.4	0	100
MbraAG-M26-3	egg	0	100	0	100	29.1	70.9	0	100
MbraAG-M26-4	egg	20.4	79.6	15.4	84.6	26.4	73.6	0	100
MbraAG-M26-5	egg	24.4	75.6	10.1	89.9	27.8	72.2	0	100
MbraAG-M26-6	egg	29.1	70.9	20.7	79.3	34.7	65.3	0	100
MbraAG-M26-7	egg	27.9	72.1	0	100	29.6	70.4	0	100
MbraAG-M26-8	egg	25.3	74.7	0	100	30.2	69.8	0	100
MbraAG-P6-pleopod	female	28.7	71.3	0	100	30.5	69.5	100	0
MbraAG-P6-mouth-part	female	0	100	0	100	21.6	78.4	100	0
MbraAG-P6-muscle	female	0	100	0	100	0	100	100	0
MbraAG-P6-setae	female	32.5	67.5	26.1	73.9	30.1	69.9	100	0
MbraAG-P6-1	egg	25.1	74.9	0	100	28.8	71.2	0	100
MbraAG-P6-2	egg	27.6	72.4	30.5	69.5	34.0	66.0	0	100
MbraAG-P6-3	egg	19.8	80.2	0	100	24.8	75.2	0	100
MbraAG-P6-4	egg	0	100	0	100	0	100	0	100
MbraAG-P6-5	egg	0	100	0	100	0	100	100	0
MbraAG-P6-6	egg	18.2	81.8	0	100	23.6	76.4	0	100
MbraAG-P6-7	egg	0	100	0	100	33.7	66.3	100	0
MbraAG-P6-8	egg	0	100	0	100	22.8	77.2	100	0
MbraAG-P6-9	egg	43.1	56.9	0	100	48.6	51.4	0	100
MbraAG-P6-14	egg	23.8	76.2	0	100	39.3	60.7	0	100
MbraAG-P6-15	egg	22.5	77.5	0	100	25.5	74.5	0	100
MbraAG-P7-pleopod	female	0	100	0	100	22.6	77.4	100	0
MbraAG-P7-1	egg	34.7	65.3	24.7	75.3	39.0	61.0	0	100
MbraAG-P7-3	egg	48.7	51.3	42.8	57.2	64.9	35.1	0	100
MbraAG-P7-5	egg	37.6	62.4	46.7	53.3	49.8	50.2	0	100
MbraAG-P7-6	egg	32.2	67.8	18.9	81.1	64.6	35.4	0	100
MbraAG-P7-7	egg	0	100	0	100	26.0	74.0	0	100
MbraAG-P7-8	egg	44.5	55.5	0	100	60.9	39.1	0	100
MbraAG-3IMDB-gonad	female	0	100	0	100	16.2	83.8	79.8	20.2
MbraAG-3IMDB-gill	female	18.6	81.4	0	100	18.5	81.5	100	0
MbraAG-3IMDB-muscle	female	0	100	0	100	0	100	100	0
MbraAG-4D2V-pleopod	female	0	100	0	100	0	100	100	0
MbraAG-4D2V-gonad	female	6.7	93.3	0	100	15.4	84.6	82.7	17.3
MbraAG-4D2V-gill	female	57.2	42.8	50.0	50.0	66.3	33.7	100	0
MbraAG-4D2V-muscle	female	0	100	0	100	0	100	100	0
MbraAG-4D2V-heart	female	34.3	65.7	0	100	41.8	58.2	100	0
MbraAG-4D2V-stomach	female	0	100	0	100	0	100	100	0
MbraAG-4D2V-intestine	female	0	100	0	100	28.6	71.4	100	0
MbraAG-4D2V-nerve	female	0	100	0	100	0	100	100	0
MbraAG-4D2V-eye	female	82.5	17.5	0	100	71.3	28.7	100	0
MbraAG-4D2V-integument	female	0	100	0	100	32.3	67.7	100	0
MbraAG-5DV-pleopod	female	100	0	0	100	100	0	100	0
MbraAG-5DV-gonad	female	100	0	0	100	100	0	100	0
MbraAG-5DV-gill	female	100	0	0	100	100	0	100	0
MbraAG-5DV-muscle	female	100	0	0	100	100	0	100	0
MbraAG-5DV-heart	female	100	0	0	100	100	0	100	0
MbraAG-5DV-stomach	female	100	0	0	100	100	0	68.0	32.0
MbraAG-5DV-intestine	female	100	0	0	100	100	0	100	0
MbraAG-5DV-nerve	female	100	0	0	100	100	0	100	0
MbraAG-5DV-eye	female	100	0	0	100	100	0	62.5	37.5
MbraAG-5DV-integument	female	100	0	0	100	100	0	100	0
MbraAG-5DV-hepatopancreas	female	100	0	0	100	100	0	100	0

Proportions were calculated by dividing the height of each peak in the electropherogram by the summation of the height of both peaks. Samples tagged as “pleopod”, “mouth-part” and “muscle” correspond to pleopod setae, endites and muscle from pereiopod, respectively.

Regarding the amplifications of COI gene from different tissues of four females, electropherogram analyses showed that the presence of ambiguous sites is very variable among different tissues. Thus, while in the female MbraAG-4D2V the heteroplasmy is maintained in most of the tissues (except pleopod setae, pereiopod muscle, stomach and nerve), in the female MbraAG-5DV it is only observed in two of the ten tissues analysed (stomach and eye) (Table 5).

For the female P6, the muscle is homoplasmic for the 16S gene and the COI gene and the pleopod and periopod setae are heteroplasmic for both genes. Regarding the mouth-parts, heteroplasmy was detected only for the mtDNA of the COI gene. *A priori*, one would expect that the samples of pleopods and mouth-parts are mostly composed of muscular tissue. However, when sampling the pleopods, we collected only the setae that cover these structures. For this reason, it makes sense that the result obtained for the pleopods would be the same as for the pereiopod setae. On the other hand, the endite is a multi-tissular structure, so, after homogenization, a mixture of mtDNA from different tissues (homo or heteroplasmic in the case of the COI gene) could be present in sample.

## Discussion

Until now, a low incidence of heteroplasmy was recorded in animals except for mussels. However, Williams *et al*. [13] had detected high levels of heteroplasmy in the blue crab *Callinectes sapidus*, identifying a high number of haplotypes never before found in metazoans [13]. In that work, the researchers propose several explanations to this phenomenon (Nuclear Mitochondrial DNA segments or NUMTS, paternal leakage, accumulated mutations and replication errors) without opting for one. In our study, the analysis of the mtDNA of a large offspring for seven spider crab females (Williams *et al*. [13] only analysed one male, one female and one megalopa) allows us to discard several hypotheses of the origin of heteroplasmy in *M*. *brachydactyla*, until reduce them to one option. In the case of this species, the high number of heteroplasmic cells per individual/tissue makes it possible to detect this phenomenon by conventional PCR. However, new techniques such as qPCR (quantitative Polymerase Chain Reaction) and ARMS-qPCR (Amplification Refractory Mutation System-quantitative PCR) are now available to detect very low levels of heteroplasmic cells [7, 8].

Electropherogram reads are conditioned by the quality and efficiency of the PCR. This is associated with the hybridisation primer/target and the efficiency of Taq polymerase among other circumstances. Contamination of samples or PCR products is a factor to have into consideration. For this reason, DNA extraction was repeated in many cases, particularly in those where double peak was detected, as it was mentioned in material and methods and results sections. However, no different results were obtained. It should be noted that double peaks only affect two specific positions in 16S rDNA and four in COI and that none of these COI positions are involved in stop codons. The similarity between the sequences obtained and the sequences available on databases indicates that it is *M*. *brachydactyla* DNA in all cases.

Once the contamination has been discarded as the cause of the double peaks, there are two different possibilities that can lead to an erroneous interpretation of the electropherograms. Firstly, the existence of NUMTs. In this sense, there are references to the presence of COI-like sequences in many crustaceans, for instance in krill, crabs, amphipods, crayfish, squat lobsters, shrimps, isopods, barnacles and copepods [64, 65]. Assuming that they were, these nuclear sequences would have Mendelian inheritance, which would imply that all the cells of an individual should have them. This hypothesis would not explain why some of the analysed tissues have a duplicate COI sequence and why others do not. For example, for COI, the female MbraAG-4D2V does not present double peaks in the pleopod setae, pereiopod muscle, stomach and nerve tissue, while they are present in gonad, gill, heart, intestine, eye and integument (Table 5). In addition, each individual could be homozygous or heterozygous for the presence of such NUMT. In this case, the female MbraAG-M26 for 16S rDNA has three combinations of sequences between positions 365 and 580 (T-A, C-T, T-T). If one of these sequences is mitochondrial, the other two would be nuclear, therefore, all her eggs should carry two combinations (one mitochondrial and one maternal NUMT) and this is not detected (Table 2). If a female were homozygous for the NUMT all descendants would have to present double peak (one mitochondrial and one NUMT) which does not happen in many cases (16S rDNA for females MbraAG-M26, MbraAG-P7 and MbraAG-5IN, and COI for the female MbraAG-P6). The second possibility is that there would have been a duplication in the mitochondrial genome of these sequences. This would not make sense because then all descendants of a female with a double peak should also have a double peak. In addition, we would have mitochondria in which there have been two independent duplications of the 16S rDNA gene and the COI gene (for example, the females MbraAG-M26, MbraAG-P6 or MbraAG-P7) and mitochondria in which the duplication only occurred in the 16S rDNA gene (MbraAG-10H or MbraAG-2DN).

Once these assumptions are discarded as explanations for the double peaks detected, we can conclude that we detected a true heteroplasmy in 22 adult individuals of a total of 83 studied. Heteroplasmy has been extensively studied at individual level in terms of the number of involved cells or tissues. However, it also has great importance at evolutionary level, since it affects Muller's ratchet. This theory holds that uniparental inheritance (homoplasmy) creates non-combined asexual lineages, which accumulate deleterious mutations more rapidly than their sexual counterparts [66–68]. Several mechanisms have been proposed to explain how mtDNA can overcome this evolutionary limitation such as genetic bottleneck, compensatory mutations, back mutations, mitochondrial DNA copy recruitment from the nucleus, purifying selection or recombination [69–70], being this last one the main accepted mechanism to eliminate Muller's ratchet. In a population of identical mitochondrial DNA molecules, recombination generates molecules that are identical to themselves and to parent molecules. Thus, mutation accumulation of the mtDNA molecule would be inevitable. The leakage of paternal mitochondrial DNA could have evolved to provide a means to overcome this limitation.

Due to the low frequency of recombination and *de novo* mutations in mitochondrial genome [24, 25], in our opinion, the main cause of heteroplasmy in *M*. *brachydactyla* are failures in the elimination of the male mitochondria. This fact could have two main explanations. Firstly, a special type of DUI-type inheritance as in molluscs [2, 33–36]. However, this can be discarded because in our species heteroplasmy is indistinctly detected in both males and females. Secondly, that there has been a generalized failure on the mechanisms of elimination of paternal mitochondria [3]. Until now, true biparental transmission of mitochondrial genomes from both parents (or from two distinct mating types) to zygotes as part of normal reproductive processes within a species and their persistence throughout development has not been demonstrated [23]. Nevertheless, sporadic biparental inheritance events have been documented in mammals, birds, reptiles, fish, molluscs, nematodes or arthropods [36].

One possible explanation for the retaining of male mitochondria in the offspring in *M*. *brachydactyla* is that these individuals come from interspecies or interpopulation hybridisations. In these cases, a breakdown of mechanisms to recognise and remove paternal mtDNA may occur [36, 71–74]. Several researches have been based on analysing the mtDNA of the offspring of intra- and interspecific crosses. Kondo *et al*. [75] performed crosses with different species of *Drosophila* during 10 consecutive generations (140 intraspecific and 191 interspecific crosses). The results of this study showed the presence of maternal mtDNA in the offspring of intraspecific crosses, while some individuals from interspecific crosses showed paternal leakage (three homoplasmic and one heteroplasmic individuals). Dokianakis and Ladoukakis [76] also analysed the mtDNA of the offspring of crosses between seven *Drosophila* species (31 interspecific crosses, 4 intraspecific crosses). They only detected paternal leakage in some of the hybrids, while in the descendants of intraspecific crosses the mitochondrial inheritance was strictly maternal. Kaneda *et al*. [71] conducted a similar study in mice. In crosses between individuals of the species *Mus musculus* the paternal mtDNA was detected only through the early pronucleus stage while in crosses between *M*. *musculus* and *M*. *spretus* the paternal mtDNA was detected throughout development from pronucleus stage to neonates. In other vertebrates, paternal leakage has been detected in natural hybrids of fish [77], birds [78] or amphibians [79].

The studies of Clark *et al*. [80] based on biometric characters suggested the lack of complete separation of the Atlantic and Mediterranean populations of *Carcinus*, with the potential existence of a hybrid zone between *C*. *maenas* (Atlantic) and *C*. *aestuarii* (Mediterranean). Something similar happens in the case of *M*. *brachydactyla* and *M*. *squinado*, species very close morphological and genetically [43–45, 81]. Although the distribution of *M*. *brachydactyla* is usually associated to the northeast Atlantic and the distribution of *M*. *squinado* to the Mediterranean [43–45], Abelló *et al*. [58] reported two specimens identified as *M*. *brachydactyla* in the Alborán Sea (Mediterranean Sea). Our results suggest that this gene flow could occur bi-directionally across the Strait of Gibraltar, the only area of direct contact between both species. The complex life cycles of the planktonic larval stages and benthic post-larvae determine the distribution and population dynamics of spiny spider crabs. During the planktonic larval phase, the individuals are drifted by the action of the latitudinal marine currents that are active mainly during the months of April to October. This fact could facilitate the transferring of individuals, extending the overlapping zones to northern regions. In addition to this, considerable displacements have also been described in adult states, reaching 180 km [82]. If this fact is one of the causes involved in the appearance of heteroplasmy, its incidence should be higher in regions to the south of the Iberian Peninsula than in more northerly regions, such as the French coasts or the British Islands. It should be noted that the differences in the COI mitochondrial sequences of *M*. *brachydactyla* and *M*. *squinado* range from 5 to 10 synonymal changes, so that, a priori, there should not be a problem of cytoplasmic inheritance and intragenomic conflict between the nucleus and the cytoplasm.

As for the distribution of the different mtDNA types in the embryo during development, the results obtained do not show a pattern that would allow conclusions to be drawn. Tissues that show the same mtDNA do not necessarily come from the same embryonic layer. For example, in the case of the female Mbra-AG-4D2V, tissues formed from the mesoderm show differences in their COI mitochondrial sequences: muscular tissue has no ambiguous sites, while gonadal tissue shows three heteroplasmic positions and cardiac tissue, two. Differences in mtDNA between tissues of the same individual had already been detected in mice by Shitara *et al*. [72]. They analysed 12 different tissues from 38 F1 hybrids in mouse. They detected paternal leakage in 17 of the individuals analysed, but in most of them, the paternal mtDNA was limited to one to three tissues, which varied from one mouse to another. Regarding ovarian tissue, only 6.6% of F1 hybrid females reported paternal leakage, so they concluded paternal mitochondrial DNA does not propagated stably to future generations.

If the male contribution is a sporadic and punctual phenomenon restricted to certain contact zones between congeneric species, meiotic cell drift and what happened during embryonic phases would be the cause of the differences between tissues. Mitochondrial DNA molecules pass through a genetic bottleneck process, this is, the decreasing of the number of copies, both during oogenesis and early development [83]. This process is apparently due to random partitioning of organelles containing only one or a very few mtDNA molecules and lead to rapid segregation of polymorphic mtDNA species in the progeny [84]. Its effect varies according to taxa, ranging from 1 to 349 in humans [85–87], 200 in mice [88] and from 370 to 740 in fruit fly [89]. It should also be noted that PCR amplifications are not 100% effective. This implies that by chance when any combination is in low proportion may not be detected by conventional PCR due to its low amplification. These facts could explain why in the sample MbraAG-2DN-pleopod we did not detect heteroplasmy but we detected it in one of its eggs.

To summarise, a considerable frequency of heteroplasmy were detected in the spider crab *M*. *brachydactyla*, possibly associated to hybridisation of congeneric species. Future studies in other populations of *M*. *brachydactyla* as well as the analysis of the offspring from crosses in captivity would be necessary to confirm the true origin and dimension of this finding. Nevertheless, heteroplasmy is an important aspect to take into account in studies of population management, especially in those under commercial exploitation, since effective population size could be overestimated. In species with this mitochondrial particularity, it is recommendable to use nuclear markers in molecular genetics studies. If mitochondrial markers are anyway chosen, heteroplasmy will have to be taken into consideration when the results are interpreted in order to avoid erroneous conclusions.
